# Non-susceptibility of *Enterococcus faecalis* to daptomycin detected by VITEK-2 P639 card: clinical reporting is not recommended

**DOI:** 10.1128/spectrum.02653-25

**Published:** 2026-03-18

**Authors:** Yinting Xing, Jingjia Zhang, Long Sun, Ning Li, Huizi Wang, Yanling Zhang, Xinmiao Jia, Hongli Sun

**Affiliations:** 1 Department of Laboratory Medicine, Peking Union Medical College Hospital 34732, Beijing, China; 2 Department of Laboratory Medicine, The First Affiliated Hospital of Harbin Medical University, Harbin, China; 3 Department of Clinical Laboratory, Hangzhou Women’s Hospital, Hangzhou Maternity and Child Health Care Hospital 440208 , Hangzhou, China; 4 Department of Laboratory Medicine, Luohe Central Hospital 666467 , Luohe, China; 5 Department of Laboratory Medicine, Jilin Central Hospital, Jilin, China; 6 Department of Laboratory Medicine, Ordos Central Hospital 688286 , Ordos, China; 7 Center for bioinformatics, National Infrastructures for Translational Medicine, Institute of Clinical Medicine & Peking Union Medical College Hospital, Chinese Academy of Medical Sciences and Peking Union Medical College 12501, Beijing, China; Icahn School of Medicine at Mount Sinai, New York, New York, USA

**Keywords:** daptomycin, daptomycin-nonsusceptible *Enterococcus*, broth microdilution, *Enterococcus faecalis*, antimicrobial susceptibility testing

## Abstract

We aimed to assess the comparability of daptomycin (DAP) antimicrobial susceptibility testing (AST) for *Enterococcus faecalis (E. faecalis)* using the VITEK-2 P639 card and the broth microdilution (BMD) method. The AST results of DAP of 356 *E. faecalis* were compared with those of two methods. Whole-genome sequencing was conducted, and clinical data were analyzed. The overall categorical agreement (CA) between the two methods was 28.4%, and the essential agreement (EA) was 20.2%. Through subgroup analysis, in the DAP-susceptible group by VITEK-2, the CA was 100%, and the EA was 45%. In the DAP-nonsusceptible group by VITEK-2, VITEK-2 misclassified 7.4% of the susceptible isolates as resistant and 92.2% as intermediate, with a CA of 0.4% and an EA of 12.1%. In clinical *E. faecalis*, as the MIC by VITEK-2 increased, the comparability between the two methods exhibited a significant decrease. There was no significant correlation between the results of VITEK-2 and those of Ca^2+^-deficient BMD. None of the 40-sequenced *E. faecalis* isolates carried known DAP resistance genes. Five patients (2.0%) with DNSE detected by VITEK-2 but susceptible by BMD were treated with DAP, and the anti-infective effect was associated with the application of DAP. The VITEK 2 system may overcall DAP Non-Susceptible *Enterococcus* in *E. faecalis*. We recommend a policy of not reporting DAP MICs of ≥4 µg/mL from the VITEK 2 without confirmation by a reference method, thereby preventing the unnecessary avoidance of a potentially effective therapeutic agent.

**IMPORTANCE:**

The overall CA of AST results for DAP between VITEK-2 and BMD was 28.4%, with an EA of 20.2%. In the DAP-sensitive group, CA was 100%, but in the DAP-nonsensitive group, VITEK-2 misclassified 7.4% of sensitive isolates as resistant and 92.2% as intermediate. The consistency between VITEK-2 and BMD decreased significantly as the VITEK-2 MIC value increased. No significant correlation was found between VITEK-2 results and Ca^2+^-deficient BMD. None of the sequenced *E. faecalis* isolates carried known DAP resistance genes. If the DAP MIC value ≥ 4 detected by VITEK-2 is not reported, it may lead to the loss of daptomycin susceptibility data in clinical practice and also cause bias in the national bacterial resistance detection results.

## INTRODUCTION

*Enterococcus faecalis* (*E. faecalis*) typically exhibits multidrug resistance and high pathogenicity. In cases where patients have gastrointestinal trauma, undergo surgery, have compromised immunity, or experience intestinal flora imbalance, it can cause severe nosocomial infections (1). Daptomycin (DAP), a cyclic lipopeptide antibiotic, last-line treatment adjectives, holds significant clinical value in the treatment of enterococcal bloodstream infections, infective endocarditis, and complex skin and soft tissue infections (2, 3). With the extensive application of DAP (4), the emergence of DAP Non-Susceptible *Enterococcus* (DNSE) poses a formidable challenge in clinical therapeutics (5). Currently, the global isolation rate of DNSE is on the rise worldwide, including in China, Europe, and the United States (6). Particularly in large hospitals and intensive care units (ICUs), it has emerged as a significant clinical treatment predicament (7). Hence, there is an imperative need to enhance the monitoring of DNSE and raise the requirements for the accuracy of DAP antimicrobial susceptibility testing (AST).

Many studies collectively emphasize that DAP resistance in *Enterococcus* involves complex mechanisms, including biofilm-related tolerance, alterations in nutrient transport systems, and acquisition of resistance genes (8). The increasing prevalence and complexity of resistance highlight the necessity of *Enterococcus* detection methods to guide effective therapeutic interventions (9).

Currently, in clinical laboratories, automatic susceptibility testing analyzers are commonly employed for DAP detection. Among them, the VITEK-2 from bioMérieux holds the largest market share in China. Its P639 card has not been updated since 2020, while the breakpoints for DAP against *E. faecalis* have undergone substantial updates since 2020. Additionally, there have been no recent research reports validating the ability of the P639 card to detect DAP. Our previous studies revealed that the proportion of DNSE in different types of clinical specimens ranges from 39.9% to 64.9% by VITEK-2 P639 card, far exceeding the proportion data published by European Committee on Antimicrobial Susceptibility Testing (EUCAST).

It is noteworthy that bioMérieux issued a customer letter (2020) following the breakpoint update (See Supplementary material), highlighting that due to the absence of resistant isolates or an insufficient number of resistant isolates during comparative experiments, the VITEK-2 P639 card presents limitations in detecting DAP. Consequently, the capacity of the P639 card to detect DAP resistance remains unclear. In light of this, in clinical scenarios where DNSE is identified and there are no Clinical and Laboratory Standards Institute (CLSI)-recommended broth microdilution (BMD) reagents for revalidation, the DAP result cannot be reported, thereby causing complications in the clinical application of DAP. Therefore, there is an urgent need to employ BMD to verify the comparability and error rate of the current VITEK-2 DAP results and analyze the real-world clinical application circumstances to formulate a rational and accurate DAP report review protocol.

Consequently, this research endeavors to verify the DAP detection results of *E. faecalis* using the VITEK-2 P639 card by collecting *E. faecalis* isolates, validating the DAP resistance phenotype through BMD, conducting gene sequencing analysis, and summarizing the real-world treatment modalities of *E. faecalis*. It also aims to assess the diagnostic value of the P639 card for DNSE and evaluate the current medication status in the real world, thereby providing a theoretical foundation for the rational use of the VITEK-2 susceptibility testing card to report DAP susceptibility results in clinical practice.

## MATERIALS AND METHODS

### Strain preparation

The information on *E. faecalis* isolates stored at −80°C in the Department of Laboratory Medicine of Peking Union Medical College Hospital (PUMCH) was accessed. All isolates included in this study were identified as vancomycin-sensitive isolates of *E. faecalis*. Vancomycin susceptibility was determined using the VITEK 2 system with the AST-P639 card and BMD reagents. The standard strains ATCC29213 and ATCC29212, along with clinical isolates, were cultured onto blood agar plate medium and incubated at 37°C for 18–24 h to obtain the first-generation pure isolates. Those isolates identified as *E. faecalis* by Smart MS 5020 mass spectrometry (Software: DL Mass Software, Zhuhai Dier Bioengineering Co., Ltd.) were further cultured to obtain the second-generation isolates for AST.

The inclusion criteria for isolates were as follows: The specimen types from which the isolates originated included urine, excreta, rectal swabs, vaginal secretions, and blood; *E. faecalis* was isolated from clinical samples between April 2022 and November 2024, with the isolates remaining in the storage system. The DNSE isolates screened by VITEK-2 and the DAP-susceptible *E. faecalis* (DSE) were included at a ratio of 2:1. The exclusion criteria were as follows**:** isolates that were not identified as *E. faecalis* by mass spectrometry after storage.

### AST detection

#### Preparation of BMD reagents

DAP (Patheon Italia S.P.A.) was selected to prepare BMD plates (unit: μg/mL). The concentrations were 0.004 to 128 µg/mL, which covered the CLSI quality control (QC) range (10), the detection concentrations of clinical samples from 2022 to 2024, and the EUCAST clinical sample detection range (11). Two additional concentrations were added at both the lower and upper limits. Blank control wells and positive control wells were established (Table S1). The carrier was 100 µg/mL Ca^2+^-adjusted M-H II broth (CAMHB, BD, USA), ensuring that the final Ca^2+^ concentration reached 50 µg/mL during AST. Each well contained 50 µL of broth. The prepared reagents were stored in the dark at −80°C for subsequent use.

#### Bacterial suspension preparation and BMD-based AST

A pipette was used to transfer 2 mL of sterile saline into a sterile test tube. Several purified *E. faecalis* colonies were emulsified into the tube, and the turbidity of the bacterial suspension was adjusted to 0.5 McFarland standard (~1.0 × 10⁸ CFU/mL). A volume of the 0.5 McFarland suspension was quantitatively transferred using a pipette into noncalcium-adjusted broth at a 1:100 dilution ratio. The diluted bacterial suspension was thoroughly mixed. Subsequently, 50 µL of the diluted suspension was added to each well of the BMD plate and control wells by using an automated broth dispenser (Thermo Scientific). The plate was sealed with adhesive film, labeled, and incubated at 37°C for 16–20 hours, with stacks not exceeding three layers.

#### Reproducibility assessment of the VITEK-2 system

QC records of the VITEK-2 system from April 2022 to November 2024 were analyzed. Ten DNSE isolates identified by VITEK-2 randomly selected were re-examined to evaluate the comparability of the instrument’s detection reproducibility.

#### VITEK-2-based AST detection

Following the VITEK-2 system protocol, QC and clinical isolates were inoculated into the P639 card by professional microbiological inspectors. The instrument automatically monitored bacterial growth during incubation. The VITEK-2 DAP values were historical clinical records from April 2022 to November 2024. The DAP formulation is identical in the P639 card (available outside the US) and the corresponding AST card available within the US.

#### AST result interpretation and documentation

Results from BMD methods were subjected to a dual-review process to ensure accuracy. For BMD, MIC values were recorded only if the positive control wells demonstrated sufficient bacterial growth. According to the requirements of CLSI M100 34TH (10), the culture plate should be incubated for 16 to 20 hours to assess the DAP AST results. The MIC value was defined as the lowest drug concentration that inhibited visible bacterial growth.

### Whole-genome sequencing of clinical *E. faecalis* isolates

Genomic DNA from 40 *E. faecalis* isolates was extracted using SDS or STE methods. Purity and integrity of DNA were assessed via agarose gel electrophoresis, followed by quantification using Qubit. Qualified DNA samples were fragmented into ~350 bp segments using a Covaris ultrasonic disruptor. DNA libraries were prepared using the NEBNext Ultra DNA Library Prep Kit for Illumina (NEB, USA), involving end repair, A-tailing, adapter ligation, purification, and PCR amplification. Post-library construction, preliminary quantification was performed with Qubit 2.0, followed by dilution to 2 ng/µL. The insert size was verified using an Agilent 2100 Bioanalyzer. Libraries meeting quality criteria were quantified via Q-PCR and sequenced on the Illumina NovaSeq platform (PE150) based on the effective concentration and target sequencing depth.

### Real-World DNSE treatment analysis

Clinical data of patients with *E. faecalis* infections were collected from the electronic medical record system of PUMCH. Data included the following: Demographics: age, sex, comorbidities, and smoking/alcohol history. Clinical parameters: Invasive procedures, duration of fever, infection site, and co-pathogen detection. Treatment details: Antibiotic regimens, therapeutic efficacy, hospitalization duration, and infection outcomes. Analysis focused on treatment strategies for patients with VITEK-2-reported DNSE but BMD-confirmed susceptibility to DAP.

### Statistical analysis

Continuous variables were expressed as median (P25 and P75), and categorical data were presented as N (%). IBM SPSS Statistics 29.0 (IBM Corporation) was employed for baseline comparisons, Spearman correlation analysis, and logistic regression analysis. WHONET 2024 software was utilized to compare DAP susceptibility results between VITEK-2 and BMD, including essential agreement (EA), categorical agreement (CA), minor errors (mE), major errors (ME), and very major errors (VME). Acceptable error thresholds were defined according to CLSI guidelines: EA >= 90%, CA >= 90%, VME <= 1.5%, ME <= 3%, and mE <= 10% (12). Sequencing data were analyzed using the high-performance computing cluster of PUMCH. Graphical illustrations were generated using Photoshop 2018 (Adobe Systems Software Ireland Ltd), GraphPad Prism 8 (GraphPad Software), Microsoft Office 2019 (Microsoft Corporation), and R 4.2.0. Statistical significance was defined as a two-sided *P*-value < 0.05.

## RESULTS

### Study isolate characteristics

**Fig 1:**
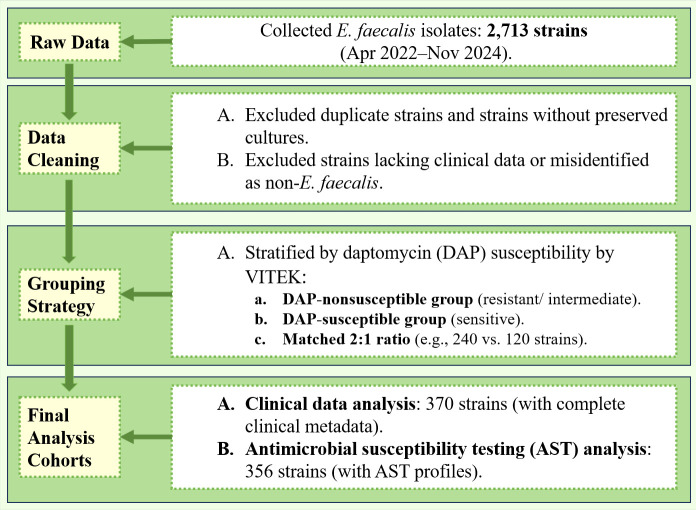
Flowchart for the screening process of *Enterococcus faecalis*.

A total of 2,713 *E. faecalis* isolates isolated from clinical specimens (urine, drainage fluid, rectal swabs, vaginal secretions, blood, etc.) at PUMCH between April 2022 and November 2024 were initially reviewed. After excluding duplicate isolates and those without preserved cultures, the remaining isolates underwent mass spectrometry confirmation for *E. faecalis* identification. Duplicates refer to isolates obtained from different anatomical sites of the same patient that are retested within a 7-day period. Following inclusion/exclusion criteria screening, 356 isolates were ultimately included for subsequent AST comparison, genomic sequencing, and clinical analysis (Fig. 1). Isolates lacking clinical metadata were further excluded during data analysis.

The study cohort had a median age of 59 years (interquartile range [IQR]: 43–69), with 48.6% being male. Complete clinical data were available for 251 patients. The median hospitalization duration was 18 days (IQR: 10–30). Among all patients, 8.8% reported smoking history, and 4.0% had alcohol consumption. Invasive procedures were performed in 78.1% of cases, with 52.2% developing postoperative fever at a median postoperative day 2 (IQR: 1–4). The median duration of fever was 3 days (IQR: 2–5). *E. faecalis*-associated infections were confirmed in 53.0% of patients (Tables S2 and S3).

### Stability assessment of VITEK-2 instrumentation

The VITEK-2 system underwent weekly QC testing using *S. aureus* ATCC 29213 and *E. faecalis* ATCC 29212, the QC strains recommended by the manufacturer (bioMérieux). For ATCC 29212, all MIC values were consistently reported as 4 µg/mL. For ATCC 29213, MIC values predominantly measured 0.25 µg/mL, with occasional results of 0.5 µg/mL, indicating stable performance within acceptable ranges and adherence to the upper limit of the QC specifications.

Ten randomly selected DNSE by VITEK-2 isolates (five with initial MIC = 8 µg/mL; five with MIC = 4 µg/mL) were retested. The CA between retested and initial results was 9/10 (90%), aligning with CLSI M100 ED34th acceptability criteria for reproducibility.

### Comparative analysis of DAP susceptibility results by VITEK-2 and BMD

In this research, the BMD results were obtained from the same batch of tests. The test results of ATCC29212 and ATCC29213 in this batch were 1 and 0.12, respectively, and the QC results were within the acceptable range. For reproducibility testing, isolates with major errors were retested from a fresh subculture on a different day, using a different lot of VITEK 2 cards and by a different technologist.

Among the 356 isolates, the CA and EA of DAP AST results by VITEK-2 and BMD were 28.4% and 20.2% respectively, revealing a considerable disparity between the two methods. The ME and mE were 5.3% and 66.3% respectively. This indicates that the overall accuracy of the test results is relatively low.

For further analysis, this study grouped the isolates based on the sensitivity to DAP as determined by VITEK-2: the DAP-S (susceptible) group and the DAP-NS (nonsusceptible) group. Among the 100 isolates in the DAP-S group, the results of VITEK-2 and BMD demonstrated a high comparability, with no errors observed, and the CA was 100% and the EA was 45%. Among the 256 isolates in the DAP-NS group, VITEK-2 misclassified 7.4% of the susceptible isolates as resistant and 92.2% as intermediate. The CA was merely 0.4%, and the EA was only 12.1%.

**Fig 2:**
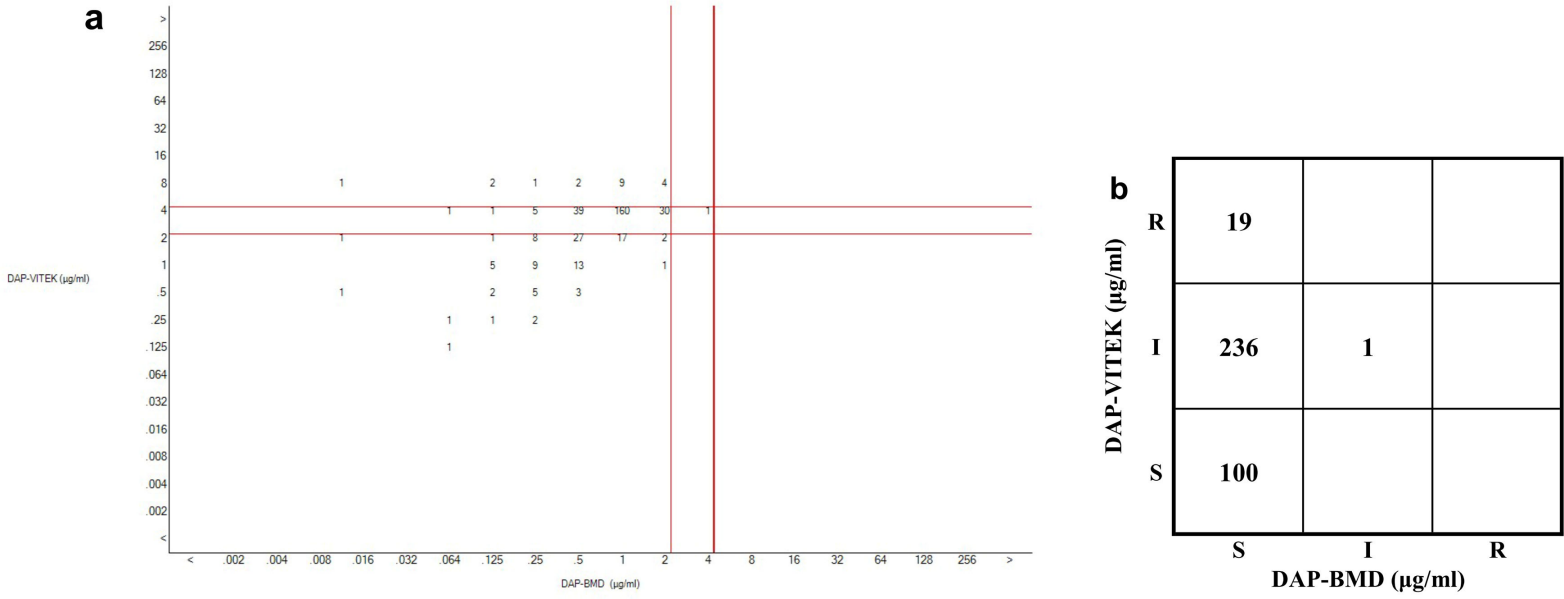
Comparison of the results of VITEK-2 and BMD for detection of DAP AST. (**a**). Scatterplot which compares DAP MIC values from VITEK and BMD methods with red cutoff lines, (**b**).Table which summarizes categorical agreement among susceptible, intermediate, and resistant classifications.

When the isolates were analyzed in separate groups based on different numerical values of VITEK-2 test results, it was observed that as the MIC value increased, the EA gradually decreased. When the DAP value was <= 1, the EA could be >50%. This suggests that the comparability between the VITEK-2 and BMD methods in the detection of DNSE is relatively low (Table 1; Fig. 2a and b).

**TABLE 1:** Comparison of AST results of DAP between VITEK-2 and BMD

Group	Strain (N)	VME (%)	ME (%)	mE (%)	CA (%)	EA (%)
DAP-T^*a*^	356	0	5.3^*b*^	66.3^*b*^	28.4^*b*^	20.2^*b*^
DAP-S	100	0	0	0	100	45^*b*^
DAP-NS	256	0	7.4^*b*^	92.2^*b*^	0.4^*b*^	12.1^*b*^
DAP-0.125^*c*^	1	0	0	0	1/1^*d*^	1/1^*d*^
DAP-0.25^*c*^	4	0	0	0	4/4^*d*^	3/4^*b*^^,*d*^
DAP-0.5^*c*^	11	0	0	0	100	72.7^*b*^
DAP-1^*c*^	28	0	0	0	100	50.0^*b*^
DAP-2^*c*^	56	0	0	0	100	33.9^*b*^
DAP-4^*c*^	237	0	0	99.6^*b*^	0.4^*b*^	13.1^*b*^
DAP-8^*c*^	19	0	100^*b*^	0	0^*b*^	0^*b*^

^*a*^ Represents all study subjects.

^*b*^ Indicates that it is beyond the acceptable error range required by the CLSI.

^*c*^ Indicates that the preceding number is the MIC value of DAP detected by VITEK-2.

^*d*^ Indicates that the number of strains is less than 10, and the percentage is not presented.

According to the results (Table 2), the MIC values detected by VITEK-2 were higher than those by BMD. Through grouping and plotting, it was evident that in the DAP-NS group, the MIC values detected by VITEK-2 were significantly higher than those by BMD. To investigate whether the higher MIC values detected by VITEK-2 were associated with the Ca^2+^ concentration, a Ca^2+^-deficient broth (BMD-Ca) with a Ca^2+^ concentration of 12.5 µg/mL was prepared for detection in this study. Through comparison, no significant linear relationship was observed between the detection results of VITEK-2 and those of Ca^2+^-deficient BMD (Table 2). Hence, the cause of the higher MIC values detected by VITEK-2 still requires further research. Isolates of DNSE detected by BMD were confirmed testing in duplicate (Table 2). For the specific distribution of AST data, please refer to Fig. S1.

**TABLE 2:** Comparison of the results of VITEK-2 and BMD for detection of DAP AST

Group	DAP-VITEK-2 M(25%, 75%)	DAP-BMD-Ca M(25%, 75%)	DAP-BMD M(25%, 75%)	*P*
DAP ALL	4.0 (2.0, 4.0)	4.0 (2.0, 4.0)^*a*^	1.0 (0.5, 1.0)	*P* < 0.0001
DAP-S	2.0 (1.0, 2.0)	4.0 (2.0, 4.0)	0.5 (0.5, 1.0)	*P* < 0.0001
DAP-NS	4.0 (4.0, 4.0)	4.0 (4.0, 4.0)^*a*^	1.0 (1.0, 1.0)	*P* < 0.0001

^*a*^ The comparison with VITEK-2 results showed that *P* > 0.05.

### Genomic analysis of DNSE *E. faecalis* detected by VITEK-2 revealed no DAP resistance genes

In this study, resistance gene analyses were performed on 40 VITEK-2 detected DNSE *E. faecalis* isolates. The bacterial resistance genes included common ones such as *AAC(6')-Ie-APH(2'')-Ia, APH(3')-IIIa, ErmA, ErmB, SAT-4, aad(6), catA8, dfrG, dfrE, efrA, efrB, emeA, lsaA, tetM, fexA, lsaE, msrC, optrA, and tet(L*). No specific resistance genes to DAP were detected.

### Summary of DAP application in patients with *E. faecalis* infection in the real world

Among the 356 patients in this study, complete case information could be obtained for 251 patients. Only five patients opted to use DAP for anti-infection therapy. All five patients had a definite diagnosis of *E. faecalis* infection. The causes of *E. faecalis* infection were postoperative anastomotic fistula, immunosuppressive status, and liver abscess. Thus, it can be observed that the patients were co-infected with multiple other pathogenic microorganisms. The antibiotics used were DAP in combination with anti-Enterobacterales antibiotics and antifungal medicines. The effectiveness of anti-infection was associated with the application of DAP (Table 3). Due to the therapeutic requirement, the doctors chose to use DAP for antibacterial treatment even without obtaining the AST results of DAP. The VITEK-2 DAP results of these five isolates were insusceptible, while the re-test results by BMD were susceptible.

**TABLE 3:** Real-world application of DAP in five patients^*a*^

AGE/Sex	Length of stay (d)	EFA infection triggers	Fever postoperative/lasts (d)	Other sites of infection	Other bacteria	Antibiotic application before AST	Antibiotic application after AST	Modified curative effect
70 /F	50	Postoperative anastomotic leakage	1/3	Bacteremia; pulmonary infection	Aviae, *C. difficile*	VA	DAP + SCF	Effective
61 /M	80	Postoperative anastomotic leakage	1/3	Duodenal fistula; abdominal infection	*Candida; S. haemolyticus*	Carpofungin + DAP + SCF	Carpofungin + DAP + SCF	Effective
59 /M	23	Postoperative anastomotic leakage	0/0	Perivalvular aortic leakage	None	CTX + DAP	CTX + DAP	Effective
59 /M	55	Immunosuppression	N/A	COVID-19 pneumonia (critical), bloodstream infection, and urinary tract infection	*P. aeruginosa, A. baumannii, and Aspergillus*	CAZ + MXF + SXT	DAP + MEM + PB	Effective
59 /M	41	liver abscess	Preoperative fever/2	Septic shock; pulmonary infection	CRKP, *E. coli*, *P. aeruginosa, Candida, and S. maltophilia*	LZD	DAP + LZD	Effective

^*a*^ VA, vancomycin; SCF, cefoperazone sulbactam; CTX, cefuroxime sodium; CAZ, ceftazidime; MXF, moxifloxacin; SXT, trimethoprim-sulfamethoxazole; PB, polymyxin B; LZD, linezolid. "Effective" represents improvement and discharge from the hospital.

## DISCUSSION

AST is the core task of clinical microbiology laboratories, and its accuracy directly influences the precise treatment of infectious diseases. The global prevalence of multidrug-resistant bacteria (such as vancomycin-resistant *Enterococcus*, VRE) has significantly augmented the clinical demand for DAP, highlighting the escalating value of AST in guiding clinical medication use (13). Nevertheless, the accuracy of the results from fully automated antimicrobial susceptibility testing systems (such as VITEK-2) for *Enterococcus* DAP remains debatable, and there is a paucity of systematic research to substantiate its accuracy in clinical decision-making (14). Concurrently, the misuse of prophylactic antibiotics (such as rifaximin) might induce cross-resistance to DAP (15), further emphasizing the exigency of optimizing AST methods. In China, tertiary grade A hospitals prevalently employ fully automated antimicrobial susceptibility analyzers (such as VITEK-2) for testing, and the data of the National Bacterial Resistance Surveillance Network predominantly rely on such devices (16). The manufacturer’s limitation acknowledges the potential for high-end variability, while the CLSI breakpoint revision heightened the clinical consequence of such variability by making any result of ≥4 µg/mL categorically “non-susceptible.”

*Enterococcus* resistance to DAP primarily arises from alterations in cell membrane charge and composition, which hinder the antibiotic’s binding and effectiveness (17). Key genetic mutations in the *liaFSR*, *yycFG*, and *cls* genes are implicated in these resistance mechanisms (18). These mutations lead to modifications in the membrane’s phospholipid content and charge, which significantly reduce DAP’s ability to insert into the bacterial membrane. Additionally, changes in cell wall thickness and surface charge further contribute to this resistance. Collectively, these adaptations diminish DAP’s capacity to disrupt the bacterial membrane, resulting in decreased efficacy against *Enterococcus* species, particularly in vancomycin-resistant *E. faecium* isolates.

The results of this study indicate that the accuracy of VITEK-2 in AST for DNSE of *E. faecalis* is suboptimal. This study reveals that the proportion of DNSE detected by clinical VITEK-2 is far higher than that by EUCAST. Given that the proportion of DAP nonsusceptible isolates reported in European countries is approximately 2% according to the EUCAST website, whereas our laboratory detected a significantly higher rate of about 50% using the VITEK-2 system, the substantial discrepancy raises concerns regarding the accuracy or comparability of the results.

The comparability of ATCC29213 between VITEK-2 and BMD methods is relatively favorable (0.25 vs 0.12), but the comparability of ATCC29212 is poor (4.0 vs. 1.0), and 4 is also the proportion with the highest detection frequency of DNSE in clinical settings. According to the data of this study, as the DAP MIC detected by VITEK-2 increases, the comparability between the results of VITEK-2 and BMD decreases.

This study discovered that regarding DNSE, the VITEK-2 DAP results did not fulfill the CLSI requirements when comparing CA, EA, ME, and mE with the standard methods (10). The CA in the DAP-S group was 100%, but within the DAP-NS group, VITEK-2 failed to meet the acceptability criteria of >= 90% agreement with BMD, supporting our decision to report only susceptible results. In terms of EA, whether in the DAP-S group or the DAP-NS group, the instrument results were significantly higher than those of the BMD method (*P* < 0.05), indicating a systematic overestimation of MIC values, which might lead to errors in subsequent studies (such as PK/PD analysis).

In this study, among the 167 VITEK-2 DNSE patients whose cases could be retrieved, it was found that five patients had a definite diagnosis of *E. faecalis* infection, and the effectiveness of the antibiotic treatment was associated with the use of DAP, further suggesting that the results of DNSE detected by VITEK-2 are not reliable. As the clinical demand for AST testing of DAP objectively exists, laboratories should establish a review method to detect and report the AST results of DAP. Among the 256 DNSE specimens tested by VITEK-2, one DNSE isolate was confirmed. This isolate was also repeatedly verified, with a minimum inhibitory concentration (MIC) value of 4, suggesting that DNSE isolates may indeed exist in clinical practice.

The VITEK 2 system may overcall DNSE in *E. faecalis*. Reporting these unverified, potentially erroneous high MICs could inappropriately steer clinicians away from using DAP. While if the DAP MIC value ≥4 detected by VITEK-2 is not reported, it may lead to the loss of DAP susceptibility data and also cause bias in the national bacterial resistance detection results. Therefore, we recommend a policy of not reporting DAP MICs of ≥4 µg/mL from the VITEK 2 without confirmation by a reference method, thereby preventing the unnecessary avoidance of a potentially effective therapeutic agent. What is more important is that laboratories should establish a DAP BMD detection or re-examination protocol.

Due to the extremely high requirement for Ca^2+^ concentration in DAP susceptibility testing, currently, no DAP E-TEST or KB reagents are available in our laboratory. In accordance with the methodologies of the CLSI and the International Organization for Standardization (ISO), the recommended reference approach for DAP AST is via the BMD method. The EUCAST (http://www.eucast.org) currently does not offer specific guidelines for DAP AST. Nevertheless, laboratories can establish their own DAP BMD reagents in accordance with the requirements of CLSI M100. When developing DAP BMD reagents in the laboratory, reagent stability is a crucial factor in ensuring the accuracy and precision of clinical outcomes. Previous studies have indicated that DAP demonstrates strong stability. Diagnostic-grade DAP powder is soluble in water and can remain stable for up to 12 hours at room temperature when shielded from light, for up to 48 hours at 2 to 8°C, and for several months to a year when stored at −20°C to −70°C (19, 20).

This study conducted an initial exploration into the reasons for the elevated DAP results from VITEK-2. The comparison results between VITEK-2 and Ca^2+^-deficient BMD did not reveal a significant correlation, suggesting that the elevated instrument results have no significant association with Ca^2+^. Through sequencing analysis of clinical samples, no known DAP resistance genes were identified in VITEK-2 DAP nonsusceptible but BMD-susceptible isolates, further indicating that the elevated instrument results are not related to the known DAP resistance genes. The specific cause of the elevated MIC values of DAP detected by VITEK-2 requires further investigation.

A study (21) has found imprecision in DAP MIC determination for *E. faecium*. However, that study involved a limited sample size of only 40 cases and was specifically focused on *E. faecium*. Usually, the findings regarding precision cannot be directly generalized to *E. faecalis* when interpreting susceptibility test results. This potential limitation was taken into account in the present study. Accordingly, during the initial development of the BMD method, inter-batch precision was assessed using the QC strains ATCC 29212 and ATCC 29213. The results demonstrated a 100% comparability rate across three batches. Furthermore, all samples in this study were tested within a single batch, and the sample size was substantially larger, which helped minimize random errors and enhance the accuracy of the results.

The isolate sequencing referred to in this article involves second-generation whole-genome sequencing. Additionally, we conducted a focused analysis of the *liaFSR* (22) system associated with DAP resistance and observed that the tested *E. faecalis* isolates did not express *liaFSR*. Consequently, the accuracy of the VITEK-2 detection results, which indicated insensitivity, may be questioned.

The BMD batch testing in this study was carried out by five qualified clinical microbiology professionals in 1 day. All microbiology personnel were trained and certified staff members. They prepared bacterial suspensions with a turbidity of 0.5 McFarland units and subsequently diluted these suspensions into the designated broths. The diluted broths were then dispensed into BMD 96-well plates using an automated broth dispenser (Thermo Scientific) that had undergone calibration and performance validation. The use of this calibrated equipment helped minimize random errors associated with manual handling.

The customer letters about DAP detection, originally issued in 2020, were reissued in 2023 without specifying the number of drug-resistant isolates. As a result, this study may represent the most comprehensive report to date that includes a significant number of VITEK-2 test-insensitive isolates.

This study still has certain limitations. Although the influence of Ca^2+^ concentration was excluded by comparing the results of the VITEK-2 system and Ca^2+^-deficient BMD, other potential influencing factors, such as medium composition and incubation conditions, still need to be further explored. The number of cases of clinical application of DAP for *E. faecalis* treatment that could be retrieved in this study was limited and did not reach the quantity for statistical analysis. Additionally, the number of included isolates was also limited. Further studies should increase the sample size to enhance the generalizability of the research findings.

### Conclusion

The VITEK-2 system exhibits inadequate accuracy in determining the DNSE, with a high false-positive rate for nonsusceptible results. This may potentially lead to clinical misjudgments and excessive avoidance of the use of DAP, influencing the analysis of large-scale bacterial resistance monitoring results. The BMD method is the recommended one. It is advisable for laboratories to establish a BMD re-examination procedure or alternative schemes to ensure the accuracy of the outcomes. The stability of self-developed BMD reagents for DAP is influenced by storage conditions, and it is essential to strictly adhere to the standards of avoiding light and low-temperature storage to improve the stability of reagents.

## SUPPLEMENTAL MATERIAL

Supplemental tables, figures, and material spectrum.02653-25-s0001.docx Tables S1 to S3, Figure S1, and supplemental material.

ASM does not own the copyrights to Supplemental Material that may be linked to, or accessed through, an article. The authors have granted ASM a non-exclusive, world-wide license to publish the Supplemental Material files. Please contact the corresponding author directly for reuse.
