# Supplementary material for: Non-susceptibility of Enterococcus faecalis to daptomycin detected by VITEK-2 P639 card: clinical reporting is not recommended
Source: Microbiol Spectr. 2026 Mar 18;14(4):e02653-25. doi: 10.1128/spectrum.02653-25 (PMC13055359; doi:10.1128/spectrum.02653-25)

**Supplementary materials**

**Contents**

[**Supplementary Table 1. List of DAP QC range, 2022-2024 clinical sample test range, EUCAST sample test range (unit: μg/mL)** 1](#_Toc204016768)

[**Supplementary Table 2. The laboratory test results and other measurement data of the patients in this study.** 1](#_Toc204016769)

[**Supplementary Table 3. Complete clinical data of 251 patients.** 1](#_Toc204016770)

[**Supplementary** **Figure 1. Comparison of the results of VITEK-2 and BMD for detection of DAP AST.** 4](#_Toc204016771)

[**Supplementary Material 1. bioMérieux’s customer letter (2020)** 5](#_Toc204016772)

# **Supplementary Table 1. List of DAP QC range, 2022-2024 clinical sample test range, EUCAST sample test range (unit: μg/mL)**

| **Antibiotic** | **QC range** | **Previous clinical sample results** | **EUCAST** | **20×liquor concentration** |
| --- | --- | --- | --- | --- |
| DAP | 0.12-4 | 0.12-8 | 0.016-32 | 5120 |

# **Supplementary Table 2. The laboratory test results and other measurement data of the patients in this study.**

| **Items** | **Case (N)** | **Min** | **Max** | **M (P25, P75)** |
| --- | --- | --- | --- | --- |
| AGE | 356 | 0.00 | 102.00 | 59 (43, 69) |
| Length of stay (d) | 251 | 0.00 | 330.00 | 18 (10, 30) |
| Fever day post-surgery | 126 | 0 | 69 | 2 (1, 4) |
| Fever lasts (d) | 149 | 0 | 60 | 3 (2, 5) |
| PLT | 239 | 1.00 | 977.00 | 209.00 (144.00, 278.00) |
| WBC | 240 | 0.30 | 264.00 | 9.50 (6.20, 14.67) |
| LYM | 240 | 0.00 | 5.98 | 0.90 (0.56, 1.39) |
| MONO | 240 | 0.00 | 2.60 | 0.46 (0.29, 0.72) |
| NEU | 240 | 0.07 | 54.72 | 7.67 (4.33, 12.52) |
| ESO | 240 | 0.00 | 2.90 | 0.04 (0.01, 0.11) |
| BASO | 240 | 0.00 | 0.53 | 0.02 (0.01, 0.03) |
| CRP | 189 | 0.58 | 336.80 | 100.80 (43.15, 159.37) |
| FIB | 201 | 0.98 | 31.00 | 4.21 (2.90, 5.06) |
| D-DIMER | 194 | 0.15 | 179.71 | 3.42 (1.40, 6.51) |
| PCT | 159 | 0.08 | 69.00 | 0.41 (0.18, 1.90) |
| U WBC | 29 | 15.00 | 500.00 | 70 (15, 125) |
| ESR | 23 | 2.00 | 112.00 | 35 (18, 66) |
| AB WBC | 7 | 63.00 | 14342.00 | 290 (102, 7624) |

# **Supplementary Table 3. Complete clinical data of 251 patients.**

| **Items** | **Case (N)** | **SUB Items** | **N（%）** |
| --- | --- | --- | --- |
| Sex | 356 | Male | 173 (48.6) |
| Smoke | 251 | Y | 22 (8.8) |
| Drinking | 251 | Y | 10 (4.0) |
| Surgery or not | 251 | Y | 196 (78.1) |
| Fever post-surgery | 251 | N | 107 (42.6) |
|  |  | Y | 131 (52.2) |
|  |  | Persistent fever | 7 (2.8) |
|  |  | Preoperative fever only | 2 (0.8) |
|  |  | Intermittent fever | 3 (1.2) |
|  |  | Fever of unknown origin | 1 (0.4) |
| Site of infection | 251 | Abdominal | 51 (20.3) |
|  |  | Abdominal+fistula | 20 (8.0) |
|  |  | Abdominal+pelvic | 4 (1.6) |
|  |  | Biliary | 6 (2.4) |
|  |  | Blood | 20 (8.0) |
|  |  | Blood+abdominal | 2 (0.8) |
|  |  | Chest | 3 (1.2) |
|  |  | HIV | 1 (0.4) |
|  |  | Intestinal | 1 (0.4) |
|  |  | Liver abscess | 1 (0.4) |
|  |  | Respiratory | 6 (2.4) |
|  |  | Multi-system | 3 (1.2) |
|  |  | Urine | 16 (6.4) |
|  |  | Newborn | 2 (0.8) |
|  |  | Pelvic | 15 (6.0) |
|  |  | Septic shock | 5 (2.0) |
|  |  | Skin | 5 (2.0) |
|  |  | Soft tissue | 4 (1.6) |
|  |  | N | 86 (34.3) |
| Fungi or not | 251 | Y | 22 (8.8) |
| Fungi | 251 | Candida | 15 (6.0) |
|  |  | Aspergillus | 7 (2.8) |
|  |  | None | 229 (91.2) |
| Bacteria or not | 251 | Y | 104 (41.4) |
| Bacteria | 251 | Efm | 8 (3.2) |
|  |  | Entero | 30 (12.0) |
|  |  | Aba | 2 (0.8) |
|  |  | Pae | 8 (3.2) |
|  |  | Anaerobic | 2 (0.8) |
|  |  | Other | 4 (1.6) |
|  |  | Multi-bacteria | 43 (17.1) |
|  |  | Staph | 7 (2.8) |
|  |  | None | 147 (58.6) |
| Virus or not | 251 | Y | 3 (1.2) |
| Virus | 251 | HIV | 1 (0.4) |
|  |  | HCMV | 1 (0.4) |
|  |  | HPV | 1 (0.4) |
|  |  | None | 248 (98.8) |
| Antibiotic to Gram preoperate | 251 | Antibiotic to Gram+ | 66 (26.3) |
|  |  | Antibiotic to Gram- | 115 (45.8) |
|  |  | Combined | 2 (0.8) |
|  |  | None | 68 (27.1) |
| Curative effect | 251 | Good | 727 (10.8) |
|  |  | Suboptimal | 101 (4.02) |
|  |  | Bad | 29 (11.6) |
|  |  | Untreated | 94 (37.5) |
| Antibiotic to Gram postoperate | 251 | Antibiotic to Gram+ | 132 (52.6) |
|  |  | Antibiotic to Gram- | 73 (29.1) |
|  |  | Combined | 1 (0.4) |
|  |  | None | 45 (17.9) |
| Change | 251 | Y | 115 (45.8) |
| Modified curative effect | 251 | Good | 113 (45.0) |
|  |  | Suboptimal | 57 (22.7) |
|  |  | Bad | 9 (3.6) |
|  |  | Untreated | 49 (19.5) |
|  |  | / | 23 (9.2) |
| Prognosis | 251 | Good | 184 (73.3) |
|  |  | Suboptimal | 11 (4.4) |
|  |  | Bad | 12 (4.8) |
|  |  | Die | 5 (2.0) |
|  |  | / | 39 (15.5) |

# **Supplementary** **Figure 1. Comparison of the results of VITEK-2 and BMD for detection of DAP AST.**


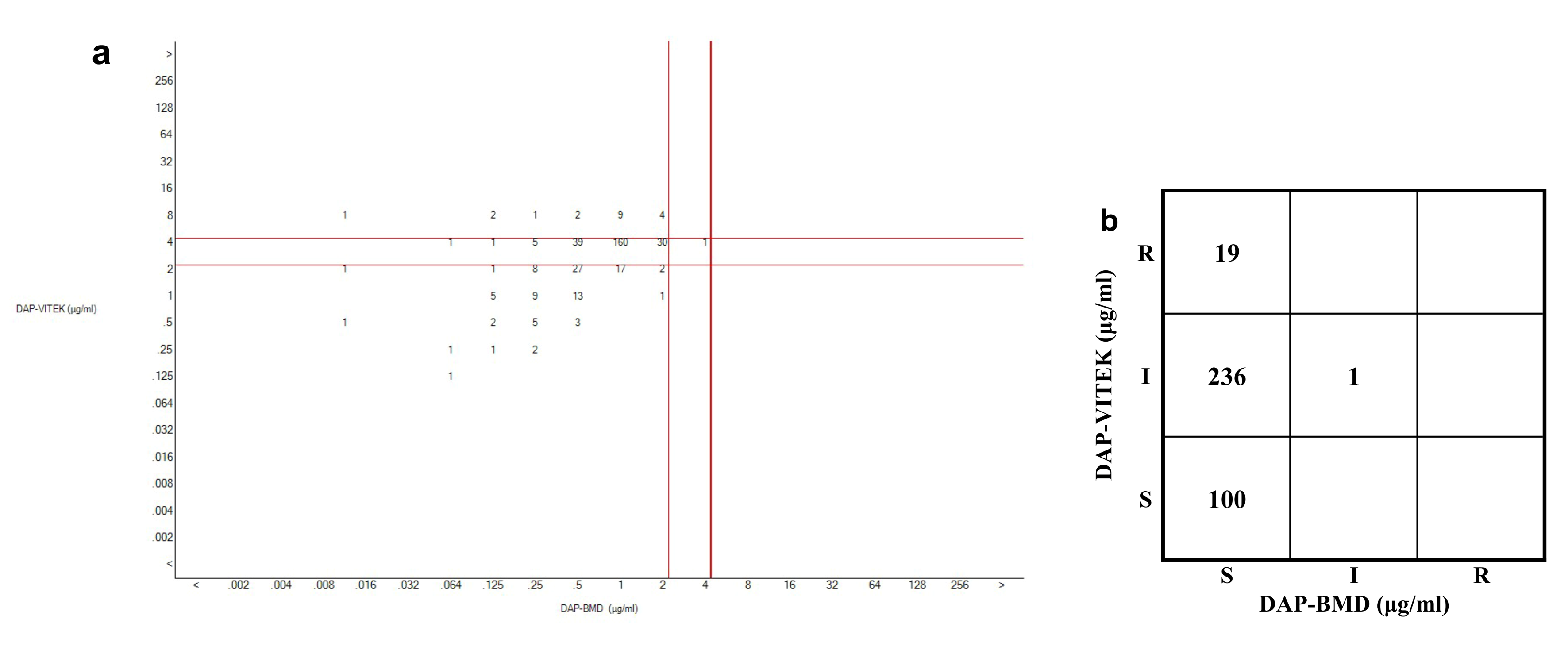


# **Supplementary Material 1. bioMérieux’s customer letter (2020)**


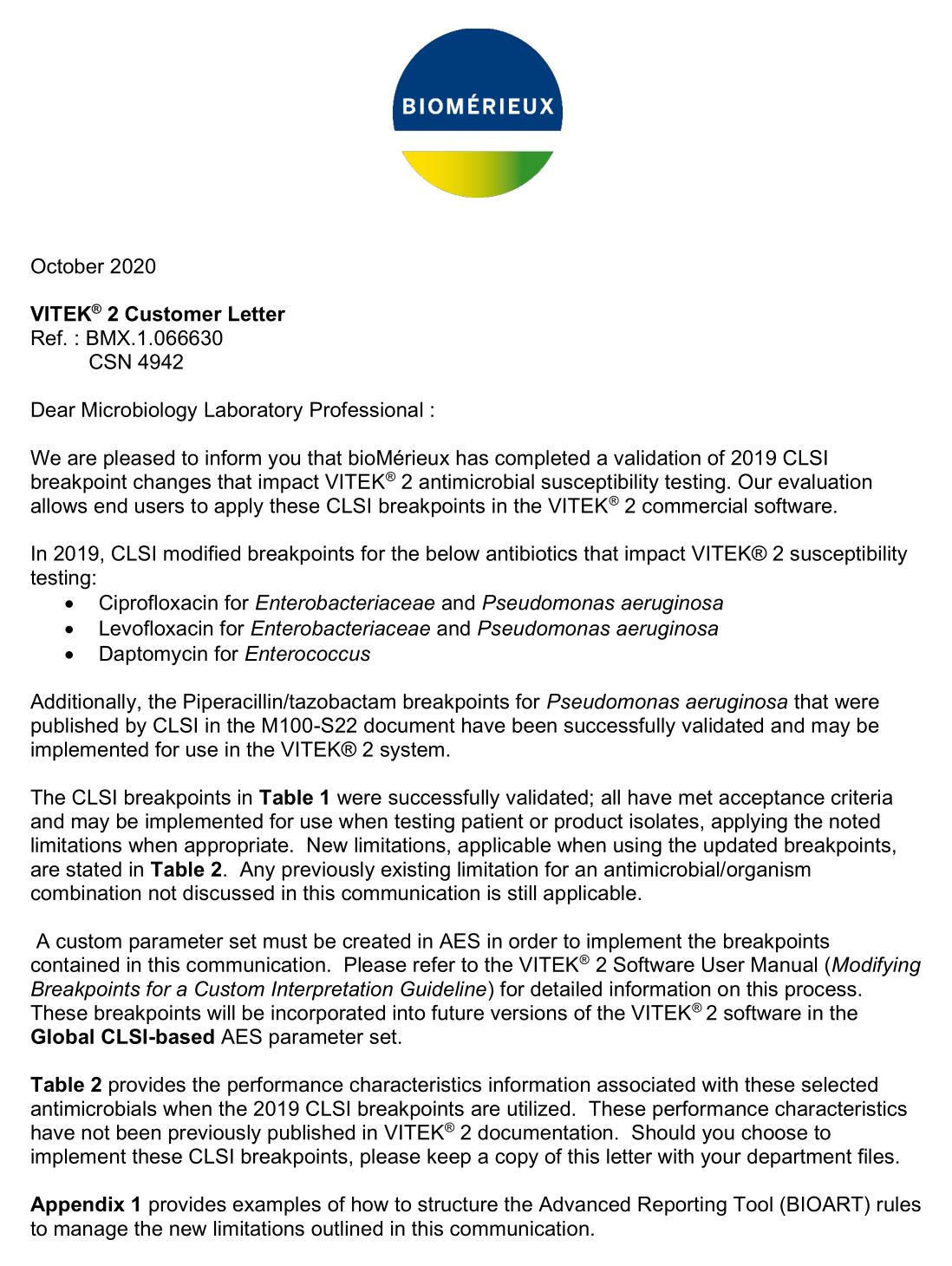


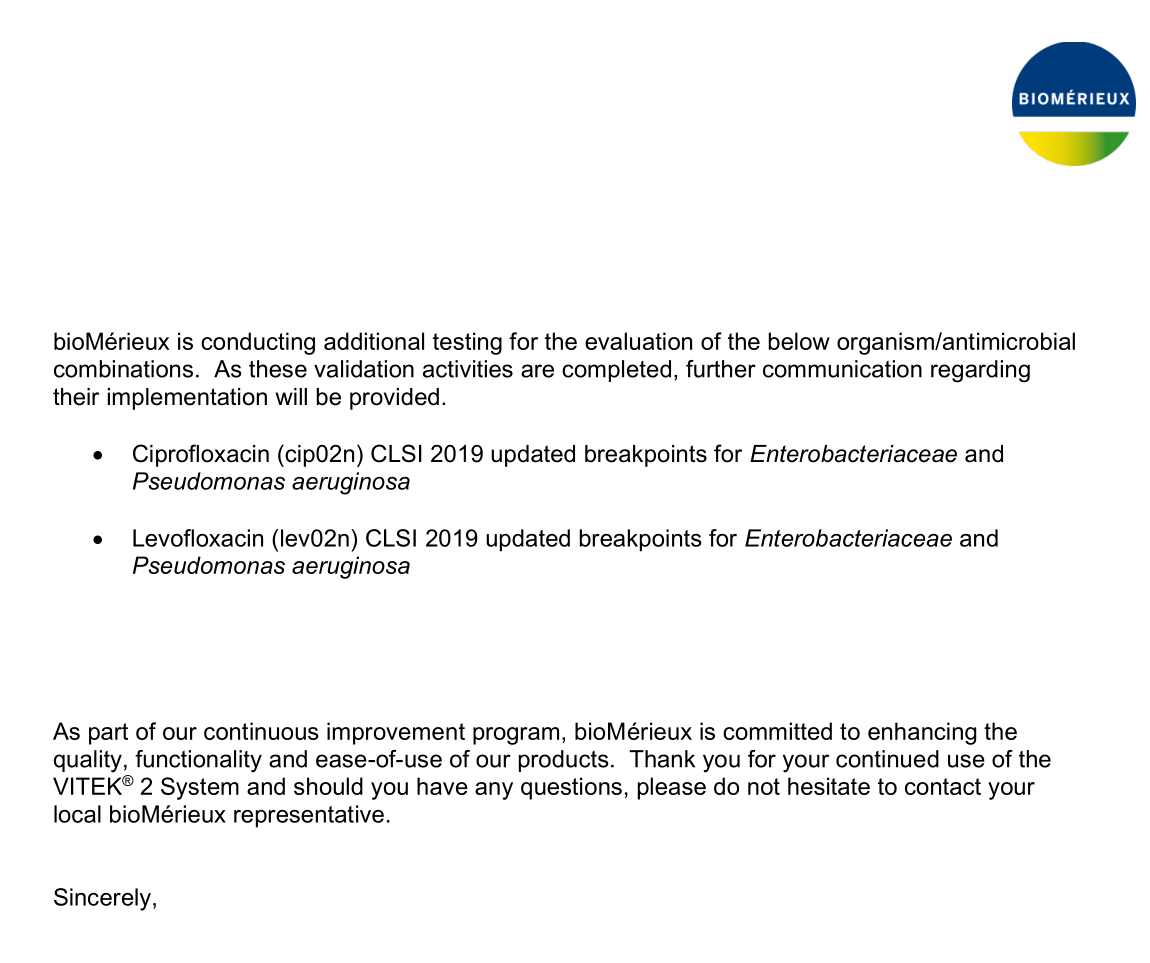


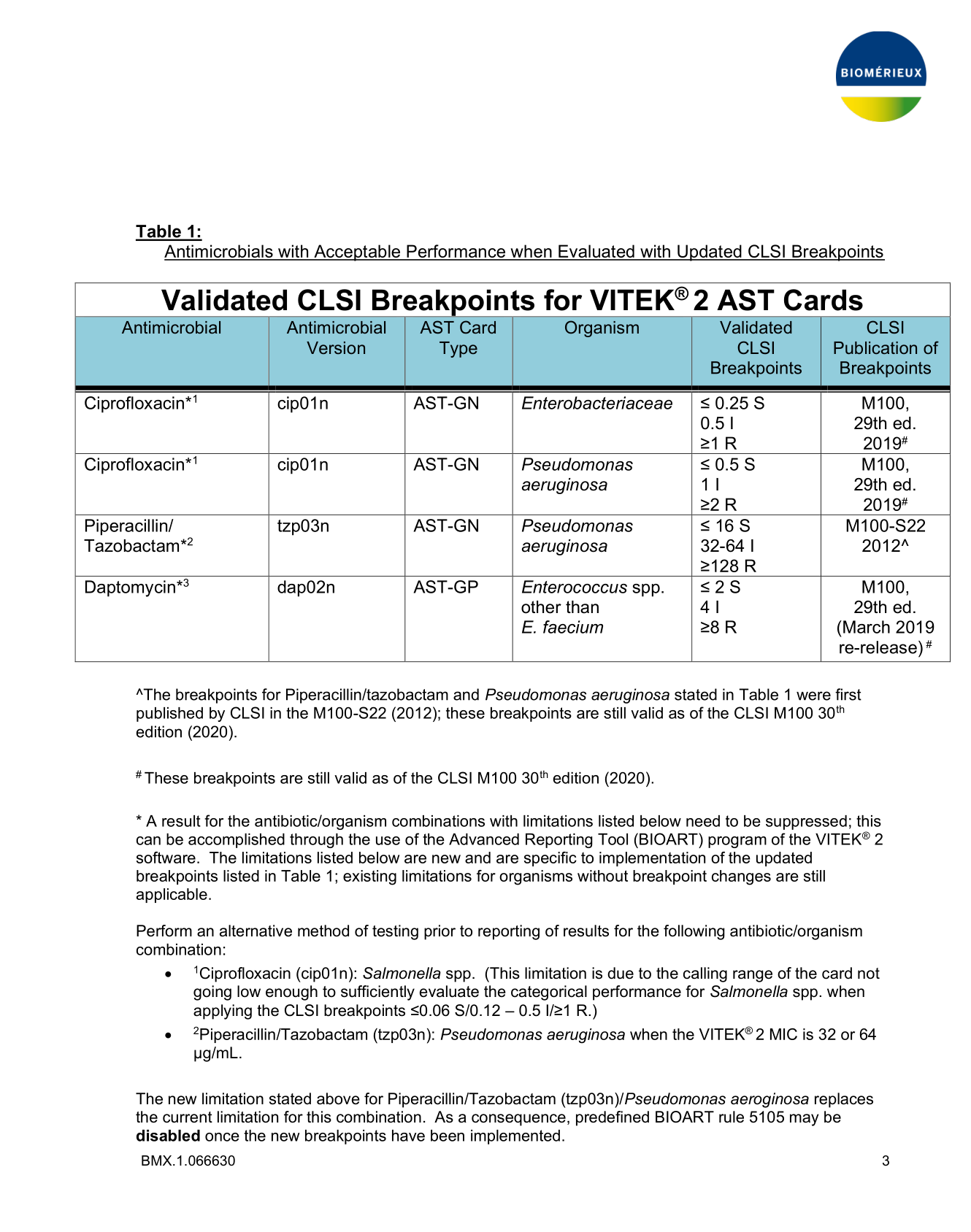


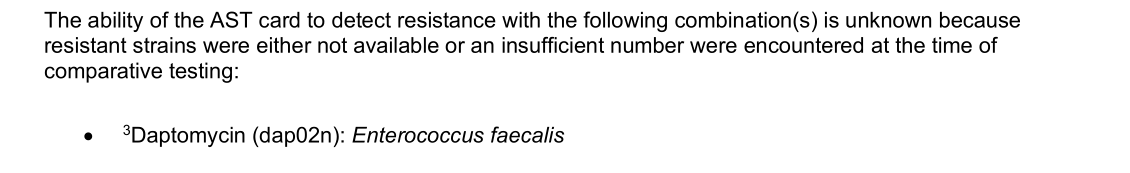


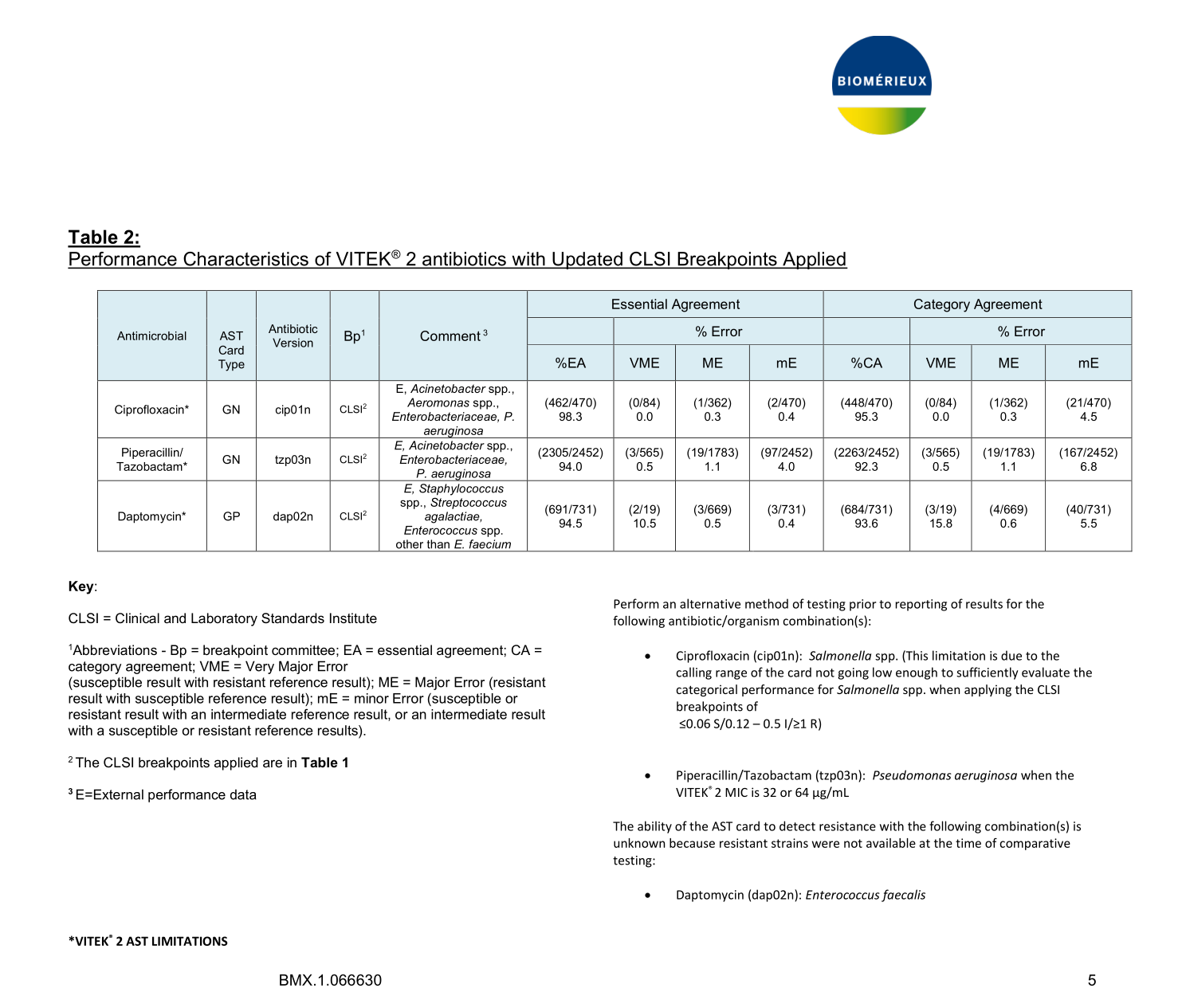


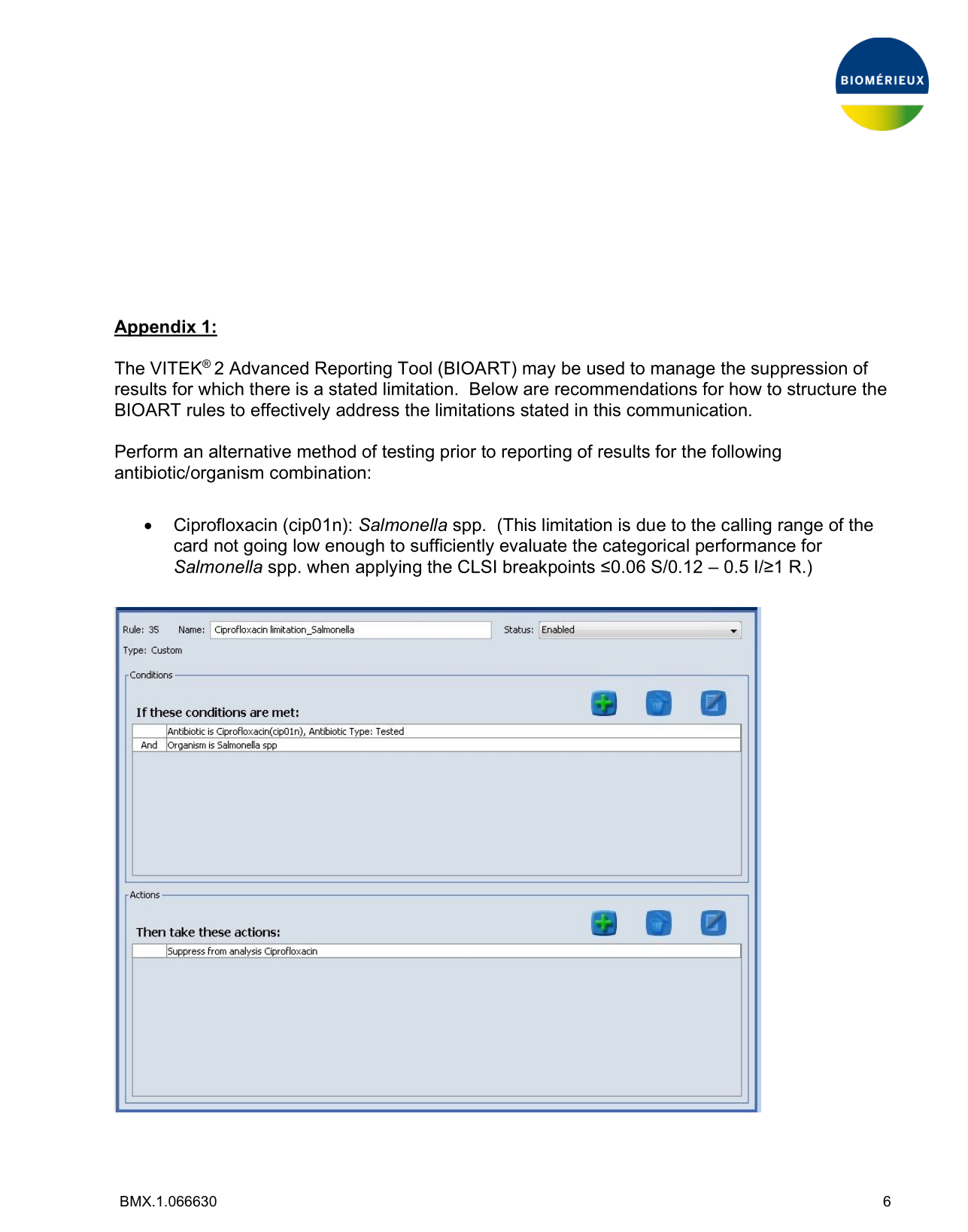


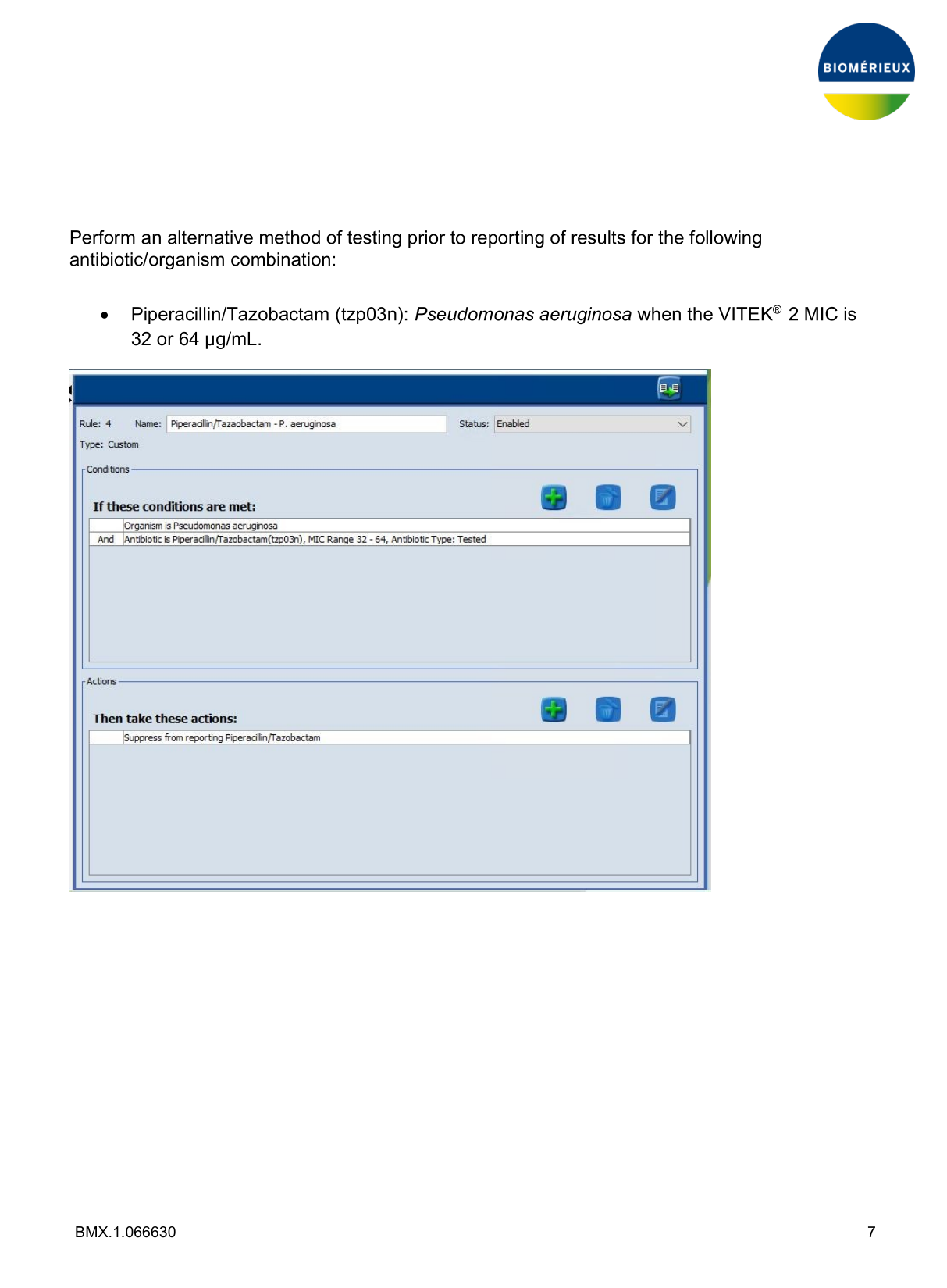


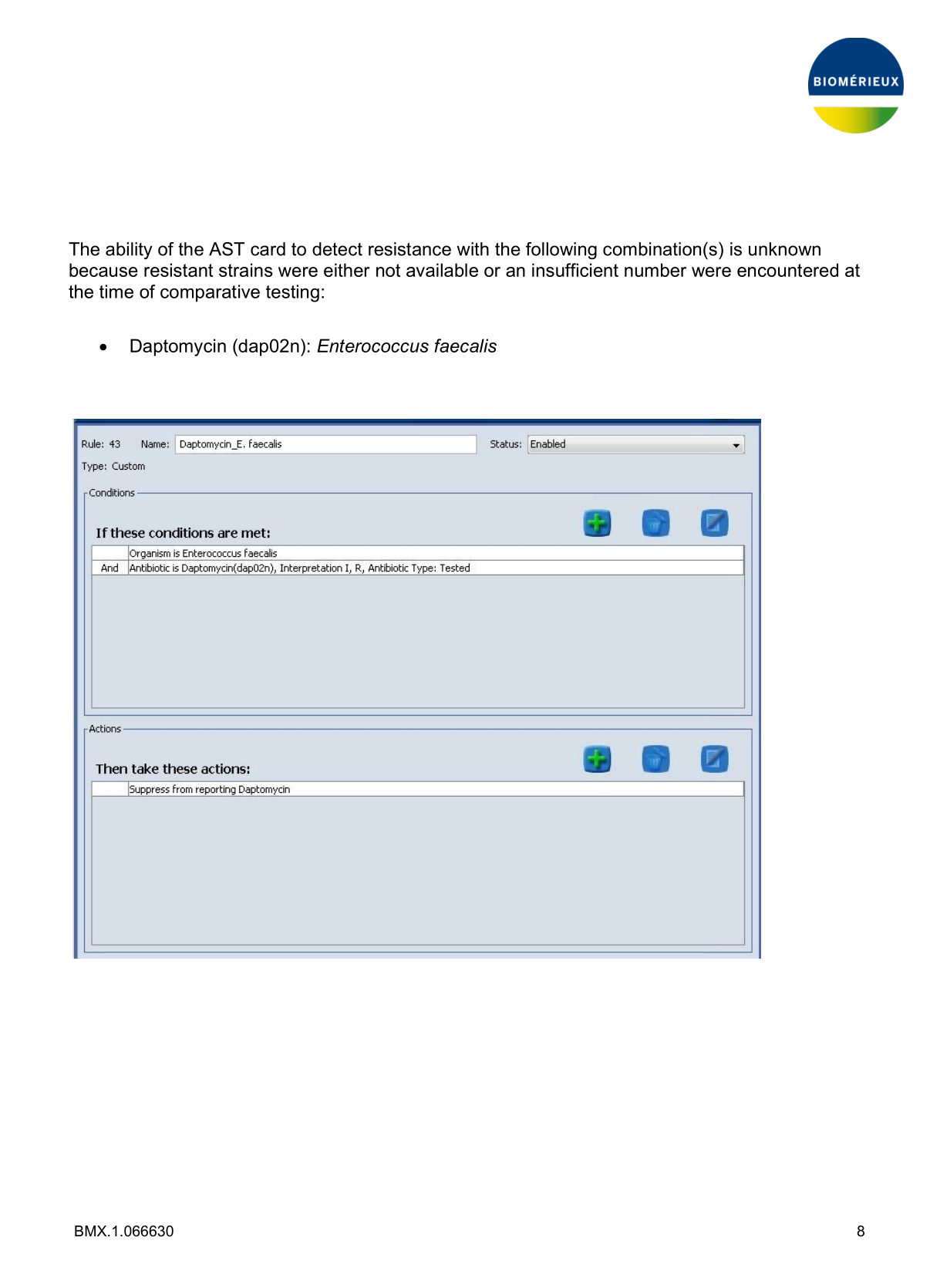

Supplement: Supplemental tables, figures, and material — Tables S1 to S3, Figure S1, and supplemental material. [file spectrum.02653-25-s0001.docx]
